# TCR repertoire dynamics and their responses underscores dengue severity

**DOI:** 10.1016/j.isci.2024.110983

**Published:** 2024-09-16

**Authors:** Kriti Khare, Sunita Yadav, Bansidhar Tarai, Sandeep Budhiraja, Rajesh Pandey

**Affiliations:** 1 Division of Immunology and Infectious Disease Biology, INtegrative GENomics of HOst-PathogEn (INGEN-HOPE) laboratory, CSIR-Institute of Genomics and Integrative Biology (CSIR-IGIB), Mall Road, Delhi 110007, India; 2 Academy of Scientific and Innovative Research (AcSIR), Ghaziabad 201002, India; 3 Max Super Speciality Hospital (A Unit of Devki Devi Foundation), Max Healthcare, Delhi 110017, India

**Keywords:** Disease, Genomics, Virology

## Abstract

Despite recognizing the immune response’s role in dengue progression, the intricate dynamics of T cell receptor (TCR) variations across DENV infection severities remain elusive. This study addresses this gap by analyzing in-house generated RNA-seq data from 112 dengue patients with varying disease severities. Our findings reveal that severe dengue patients exhibit pronounced clinical manifestations including leukopenia, thrombocytopenia, and elevated lymphocyte levels, Intriguingly, these patients also showed increased diversity in γ and δ TCR chains, unique TRGV and TRBV segment usage, and extended δ-CDR3 sequences, suggesting specialized inflammatory functions. Furthermore, mutations in the NS5 and 3′UTR regions of the dengue genome correlated with increased TRDV and TRGV chains, indicating a significant role for these mutations in the prevalence of specific TCR chains during severe infections. Overall, the study highlights the complex role of TCR repertoire in dengue pathogenesis, enhancing our understanding of TCR dynamics for future infectious diseases.

## Highlights

• Elevated γδ TCR chains in severe dengue suggest key roles in inflammation
• Severe dengue exhibits unique TRGV/TRBV usage and extended δ-CDR3 sequences
• NS5 and 3′UTR mutations correlate with increased TRDV/TRGV chain abundance
• TCR diversity and segment usage are linked to dengue severity and immune response

## Graphical abstract

**Figure:**
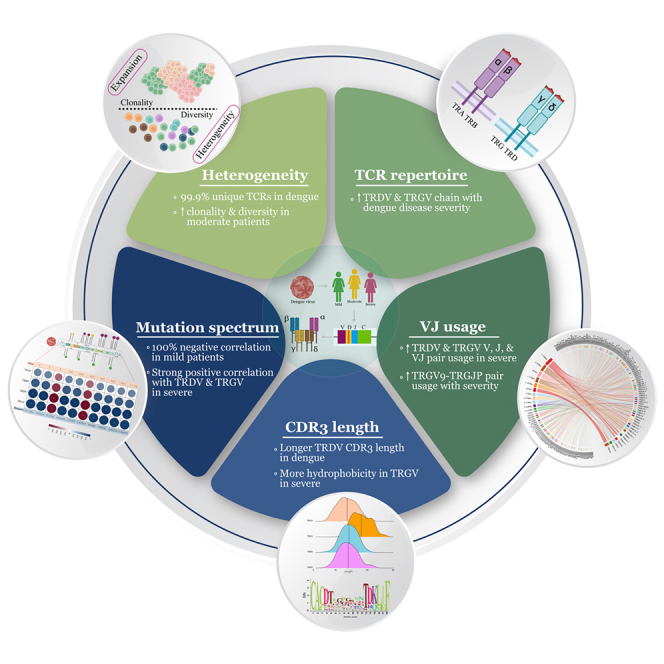

## Introduction

Dengue fever, a mosquito-borne viral infection caused by the dengue virus (DENV), is a significant public health challenge, particularly in tropical and subtropical regions.^1^ Dengue incidence has surged dramatically, with World Health Organization (WHO)-reported cases rising from 505,430 in 2000 to 5.2 million in 2019. In 2023, over 6.5 million cases and 7,300 deaths were reported across more than 80 countries, marking the highest recorded number.^2^ In India, the National Center for Vector Borne Disease Control (NCVBDC) has documented over 820,000 dengue cases and 1,100 deaths since 2018.^3^ Dengue fever, caused by any of the four main serotypes (DENV 1–4), results in regular seasonal outbreaks and epidemics, contributing to the emergence of new strains and increasingly severe disease manifestations.^4^ These frequent outbreaks exacerbate the public health burden, complicating disease management and control efforts across the country. Dengue infection can manifest in a spectrum of clinical presentations, from mild febrile illness to severe forms such as dengue hemorrhagic fever (DHF), and dengue shock syndrome (DSS).^5^ Severe dengue is often characterized by high fever, severe abdominal pain, persistent vomiting, bleeding, and plasma leakage leading to shock.^6^^,^^7^ Studies have also shown that the severity of dengue is closely correlated with the extent of leukopenia and thrombocytopenia.^8^ Patients with severe dengue often exhibit markedly lower white blood cell (WBC) and platelet counts compared to those with milder forms of the disease. These hematological abnormalities contribute to the pathophysiology of severe dengue, exacerbating the risk of bleeding, organ impairment, and shock.^9^^,^^10^^,^^11^

Previous research has highlighted the critical role of the immune system, particularly the involvement of T cell responses in disease progression.^12^^,^^13^ T cells have a complex and dynamic role in dengue virus (DENV) infection, providing both protective and harmful effects. During a secondary DENV infection with a different serotype, a phenomenon known as T cell original antigenic sin occurs,^14^^,^^15^ where cross-reactive T cells from the initial infection dominate the immune response. This predominance of low-affinity, cross-reactive memory T cells can result in poor viral control and severe disease due to the overproduction of inflammatory cytokines.^12^^,^^16^ Conversely, studies show that T cells, particularly CD8^+^ T cells, can effectively combat DENV. Certain human leukocyte antigens (HLA) alleles associated with protection against severe dengue are linked to strong, multifunctional T cell responses, indicating a crucial protective role.^17^^,^^18^ These contrasting effects highlight the complex nature of T cell responses in dengue.

For T cells to play a pivotal role in immunity and pathogenesis, it is very essential for them to identify and recognize the antigen-derived peptide as a result of infection.^19^ This facilitation of interaction between T cells and antigens is facilitated by an exceptionally diverse array of T cell receptors (TCRs).^20^ The TCR repertoire encompasses the diverse range of TCRs found within an individual’s immune system. The diversity within the TCRs arises through a process called V(D)J recombination, where gene segments encoding the variable regions of the TCR chains are nearly randomly rearranged during T cell development in the thymus.^21^^,^^22^^,^^23^ As a result, each T cell expresses a unique combination of variable gene segments, leading to a vast repertoire of TCRs capable of recognizing a wide range of antigens.^24^ In TCRαβ cells, the TCR consists of two protein chains, designated as α (alpha) and β (beta) chains, whereas in TCRγδ cells, it is composed of γ (gamma) and δ (delta) chains.^23^^,^^25^ These chains contain variable regions that are responsible for antigen recognition as well as constant regions that are involved in signaling and T cell activation. When the TCR engages with MHC-loaded peptides, it triggers T cell activation and subsequent clonal expansion.^26^ This expansion results in a shift in the repertoire specificity toward the encountered antigen.^27^ Consequently, TCR repertoires become crucial indicators of antiviral immunity, offering a functional signature of the cellular immune response.

Gaining insights into the versatile nature of TCRs and their associated immune responses during dengue virus (DENV) infection and its progression offers an insight into the functional dynamics of the host-pathogen interplay. This knowledge is crucial for deepening our comprehension of host-pathogen interactome and enhancing our ability to combat infections more effectively. In this study, we investigated TCR repertoire dynamics across three severity stages of DENV-2 infection. Marked clinical manifestations such as leukopenia, thrombocytopenia, and elevated lymphocyte levels were observed in severe dengue patients. We noted significant individual-specific TCR diversity, with severe cases exhibiting elevated γ and δ TCR chains, highlighting their role in inflammation. Additionally, severe patients showed unique TRGV and TRBV segment usage and extended δ-CDR3 sequences, suggesting specialized functions of δ-chain TCRs. Mutations in the NS5 and 3′UTR regions of the dengue genome were correlated with increased TRDV and TRGV chains, implicating these mutations in the abundance of specific TCR chains during severe infections. Together, these findings underscore the critical relevance of the TCR repertoire. This deeper understanding of TCR dynamics from an immune-genomic perspective is pivotal for advancing our knowledge of host-pathogen interactions and developing effective strategies to combat severe dengue infections.

## Results

### Clinical parameter distribution and correlation with dengue severity

**Figure 1:**
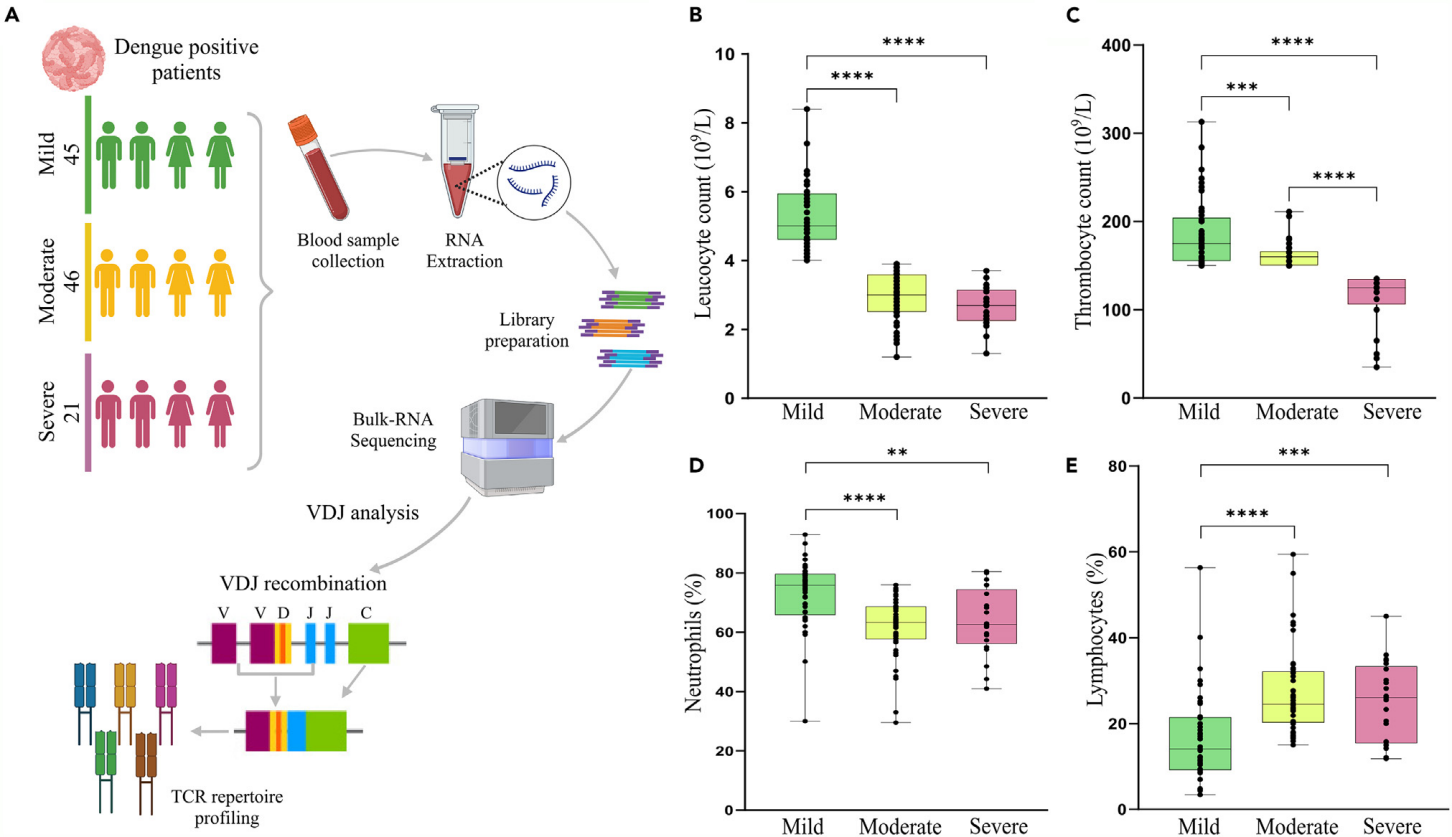
Study design, experimental workflow, and clinical demographics of dengue infected individuals with severity (A) Study design and experimental workflow including the segregation of 112 hospital admitted patients into dengue severity subgroups, and downstream TCR repertoire analysis. (B–E) Clinical and demographic parameters of the dengue severity groupings including, (B) Leukocyte count (10^9^/L), (C) Thrombocyte count (10^9^/L), (D) Neutrophil count (%), and (E) Lymphocyte count (%). Data are represented as median +/− SEM. Mann-Whitney U test was performed for calculating the statistical significance (*p* < 0.05). Significance value is denoted as ∗, where ∗ indicates *p* ≤ 0.05, ∗∗ indicates *p* ≤ 0.01, ∗∗∗ indicates *p* ≤ 0.001 and ∗∗∗∗ indicates *p* ≤ 0.0001.

The distribution and correlation of clinical parameters with dengue disease severity provide valuable insights into the diversity and prognosis of the illness. Clinical parameters encompass various physiological and laboratory measurements that reflect the patient’s health status and the severity of the disease. Hematological parameters, such as platelet count, white blood cell count, and hematocrit levels, are commonly monitored in dengue patients.^28^ Thrombocytopenia (low platelet count) and leukopenia (low white blood cell count) are frequently observed during dengue infection and may indicate disease severity, particularly in severe cases associated with dengue hemorrhagic fever (DHF) or dengue shock syndrome (DSS).^29^ According to the World Health Organization (WHO) guidelines (2009), dengue severity is classified into dengue without warning signs, dengue with warning signs, and severe dengue. However, not all patients in our study conformed precisely to these classifications, which are essential for addressing global health challenges. In our study, patients who met the criteria for dengue without warning signs and had normal platelet and leukocyte counts were classified as mild cases. Patients with dengue with warning signs were further divided into two groups: those with normal platelet counts but with leukopenia were classified as moderate cases, while those with both thrombocytopenia and leukopenia were categorized as severe cases. Although our patients did not fit neatly into the WHO-defined severity categories, we used these classifications—mild, moderate, and severe—for analytical purposes to ensure consistency and clarity in our data interpretation.^30^ Therefore, a separate group for severe dengue was not established. Groups were categorized as mild (45) (no thrombocytopenia and leukopenia), moderate (46) (no thrombocytopenia but leukopenia), and severe (21) (thrombocytopenia and leukopenia) dengue cases to examine TCR repertoire dynamics (Figure 1A). The laboratory clinical parameters (CBC and LFT) for different severity groupings in dengue patients are presented in Table 1. Few symptoms in dengue patients marked fever, vomiting, chills, abdominal pain, and body aches, were commonly reported among most patients. Additionally, some patients experienced orbital pain, rashes, sore throat, and constipation (Data S1). As expected, there was a decrease in both leukocyte and thrombocyte counts with an increase in disease severity, suggesting the presence of leukopenia and thrombocytopenia during dengue infection (Figures 1B and 1C). This significant decrease was observed in moderate and severe groups compared to mild. Significantly, we observed a decrease in neutrophils count in both moderate and severe dengue cases. These cases also showed signs of neutropenia. Interestingly, the lymphocyte count was significantly elevated in both moderate and severe groups compared to mild. These clinical parameters support the severity-based classification of dengue patients. (Figures 1D and 1E). We also investigated for any possible correlation between dengue infection and age/gender; however, there was no significant difference between age/gender and dengue disease severity subgroups (Figure S1).

**Table 1:** Distribution of laboratory blood parameters in mild, moderate, and severe dengue positive patients

Dengue clinical parameter	Mild (*n* = 45)	Moderate (*n* = 46)	Severe (*n* = 21)	*p* value	significance test
Age	27 (18–33)	26 (19–31.75)	35 (18–43)	0.139	b
Gender F|M	15|30	17|29	4|17	0.338	a
Total Leukocyte Count (TLC)	5.0 (4.6–5.9)	3.0 (2.5–3.58)	2.7 (2.3–3.1)	<0.0001	b
RBC Count	4.8 (4.51–4.99)	4.8 (4.58–5.03)	4.8 (4.51–5.58)	0.184	b
Packed Cell, Volume	41.2 (40.1–42.8)	42.3 (38.4–44.23)	43.9 (40.4–46.3)	0.756	b
Platelet Count	175 (155–202)	160 (150–165)	125 (112–135)	<0.0001	b
Neutrophils	75.9 (66.7–79.5)	63.3 (58.1–68.65)	62.6 (57.3–73)	<0.0001	b
Lymphocytes	14.1 (9.4–21.3)	24.5 (20.3–31.97)	26 (15.8–32.7)	<0.0001	b
Monocytes	9.8 (8–12)	10.0 (7.85–11.47)	9.0 (6.9–11)	0.319	b
Eosinophils	0.2 (0–0.73)	0.2 (0.1–0.88)	0.5 (0.1–1)	0.309	b
Basophils	0.5 (0.3–0.6)	0.6 (0.23–0.8)	0.5 (0.3–0.8)	0.688	b
Hb	13.6 (12.6–14.3)	13.9 (12.53–14.5)	14.1 (13.1–15.3)	0.229	b
Total Protein	7.3 (7–7.6)	6.7 (6.5–7.3)	6.7 (6.05–7.13)	0.027	b
Albumin	4.4 (4.05–4.5)	4 (3.8–4.15)	4 (3.68–4.1)	0.043	b
Globulin	3.1 (2.95–3.3)	3 (2.65–3.15)	2.7 (2.3–2.93)	0.0543	b
A.G. ratio	1.4 (1.3–1.55)	1.3 (1.2–1.5)	1.6 (1.38–1.6)	0.183	b
Bilirubin (Total)	0.7 (0.55–0.8)	0.4 (0.3–0.55)	0.8 (0.5–0.9)	0.017	b
Bilirubin (Direct)	0.1(0.12–0.17)	0.1 (0.07–0.13)	0.2 (0.13–0.23)	0.010	b
Bilirubin (Indirect)	0.6 (0.43–0.62)	0.3 (0.24–0.42)	0.6 (0.39–0.71)	0.028	b
SGOT- Aspartate Transaminase (AST)	41.0 (34.5–76)	59.0 (44–80.5)	97.5 (74.25–141.5)	0.027	b
SGPT- Alanine Transaminase (ALT)	42 (20–95)	41 (25.5–59)	66 (51.75–80.25)	0.131	b
AST/ALT Ratio	0.9 (0.82–1.73)	1.5 (1.13–1.95)	1.7 (1.2–2.17)	0.077	b
Alkaline Phosphatase	67.0 (60–89)	80.0 (59.5–100)	82.5 (59.75–124.25)	0.609	b
GGTP (Gamma GT), Serum	37 (24.5–46.5)	19 (14–41)	49 (32–82)	0.078	b

a-chi square; b-kruskal wallis.

### Differential TCR dynamics and clonotype diversity during dengue severity

**Figure 2:**
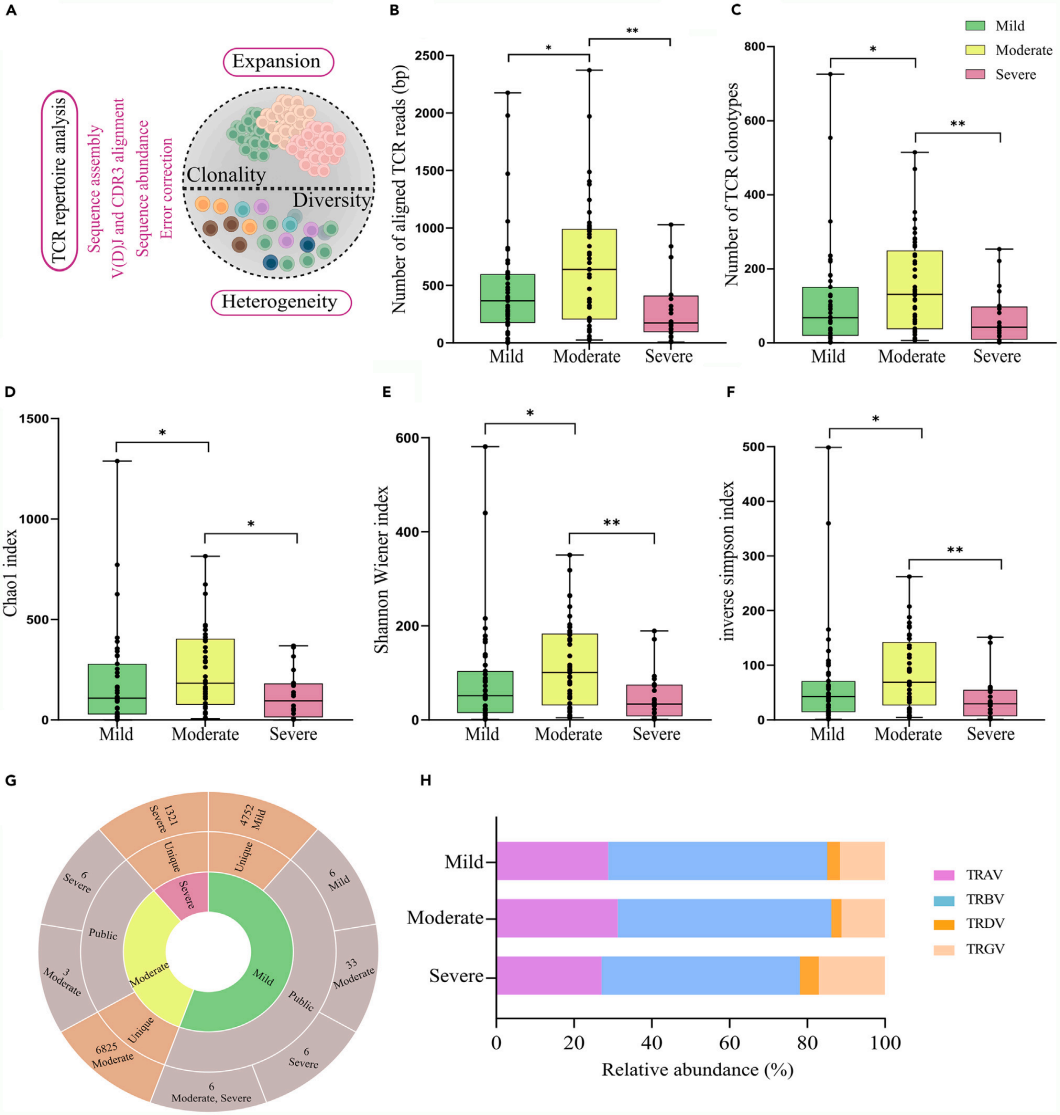
Diversity and heterogeneity of the TCR repertoire in patients with dengue infection (A) Graphical representation of clonal heterogeneity and expansion of TCR repertoire analysis. (B) Total aligned TCR reads across mild, moderate and severe patients. (C) Number of TCR clonotypes across mild, moderate and severe patients. (D–F) Clonal diversity analysis with different diversity parameters including Chao1 index, Shannon Wiener index and, inverse simpson index shows the significant differences between mild, moderate and severe patients. (G) Sunburst plot showing the number of unique and public TCR clonotype distribution across mild, moderate and severe patients. Statistical significance for number of clonotypes and diversity between the disease cohorts was calculated using Mann-Whitney U test (*p* < 0.05). (H) Relative abundance between four different TCR chains (TRAV, TRBV, TRDV, and TRDV) across the dengue disease severity. Data are represented as median +/− SEM. Fisher’s exact test was performed for calculating the statistical significance (*p* < 0.05). Significance value is denoted as ∗, where ∗ indicates *p* ≤ 0.05, ∗∗ indicates *p* ≤ 0.01, ∗∗∗ indicates *p* ≤ 0.001 and ∗∗∗∗ indicates *p* ≤ 0.0001.

Among 112 patients’ transcriptomic data analyzed using MiXCR and VDJtool to explore TCR dynamics in dengue infection (Figure 2A), 108 exhibited TCR clonotypes ranging from 1 to 727. Figure 2B shows a significant differential distribution of aligned TCR reads per million sequencing reads in the moderate patients compared to the mild and severe patients (Data S2). In total, there were 13,096 clonotypes, with 4838, 6911, and 1347 present in the mild, moderate, and severe dengue-infected individuals, respectively. The details of median and average clonotype counts for these groups are present in Data S3. Notably, a significant increase in clonotype numbers was observed in moderate patients compared to both mild and severe (Figure 2C). We also estimated the clonotype diversity indexes using various parameters such as the Shannon-Wiener index, the inverse-simpson index, the chao1 index, d50 index, chaoE index, and efron thisted indexes (Data S4). All these diversity indexes revealed a similar pattern where TCR diversity increased significantly in moderate compared to mild and severe patients (Figures 2D–2F). An increase in the number of clonotypes, along with greater diversity in the moderate stage, suggests that the moderate disease state is dynamically intermediate between the more stable mild and severe stages. This indicates that the immune response is more adaptable and varied in moderate cases, reflecting a transitional phase compared to the relatively consistent immune profiles seen in mild or severe cases.

### Association of unique TCR clonotypes and elevated δ/γ chain abundance with dengue severity

Given the immense potential diversity of TCR sequences, the sharing of TCR sequences among individuals is expected to be exceedingly rare.^31^ In our study, we investigated both shared and unique TCR clonotypes across the patients and severity subgroups. Remarkably, we found that over 99% of the clones were unique to each patient, with shared clonotypes between any two patients accounting for only 0.04–0.46% of the total clonotypes (Figure 2G). This suggests that nearly every patient possesses unique TCR clonotypes, resulting in substantial differences between the patients. Figure S2 provides detailed information on TCR clonotypes. The correlation between the number of clonotypes and the total sequencing reads from TCR was linear (R^2^ = 0.94).

About 83% of the clonotypes across the patients expressed TCRs consisting of α and β chains, and ∼17% of the detected clonotypes consisted of δ and γ chains. The relative abundance of the β chain was maximum (54%), followed by the α (29%) and γ chains (13%) in the dengue patients, while for the δ chain it was minimum (4%) (Data S5). Interestingly, the relative abundance of δ and γ chains was higher, while α and β chains were lower in severe patients than in mild and moderate patients (Figure 2H). The observed differences across the severity groups for all the TCR chains were statistically significant (Fisher exact test, *p* ≤ 0.05), except for TRBV and TRGV, which did not show significance across mild and moderate groups. Additionally, TRAV showed non-significance across mild and severe patients (Data S5). These findings imply that the increased presence of TRDV and TRGV chains correlates significantly with dengue disease severity, potentially indicating their important role and dynamic changes in response to the dengue viral infection.

### Selective V and J segment usage patterns reveal dengue severity dynamics

**Figure 3:**
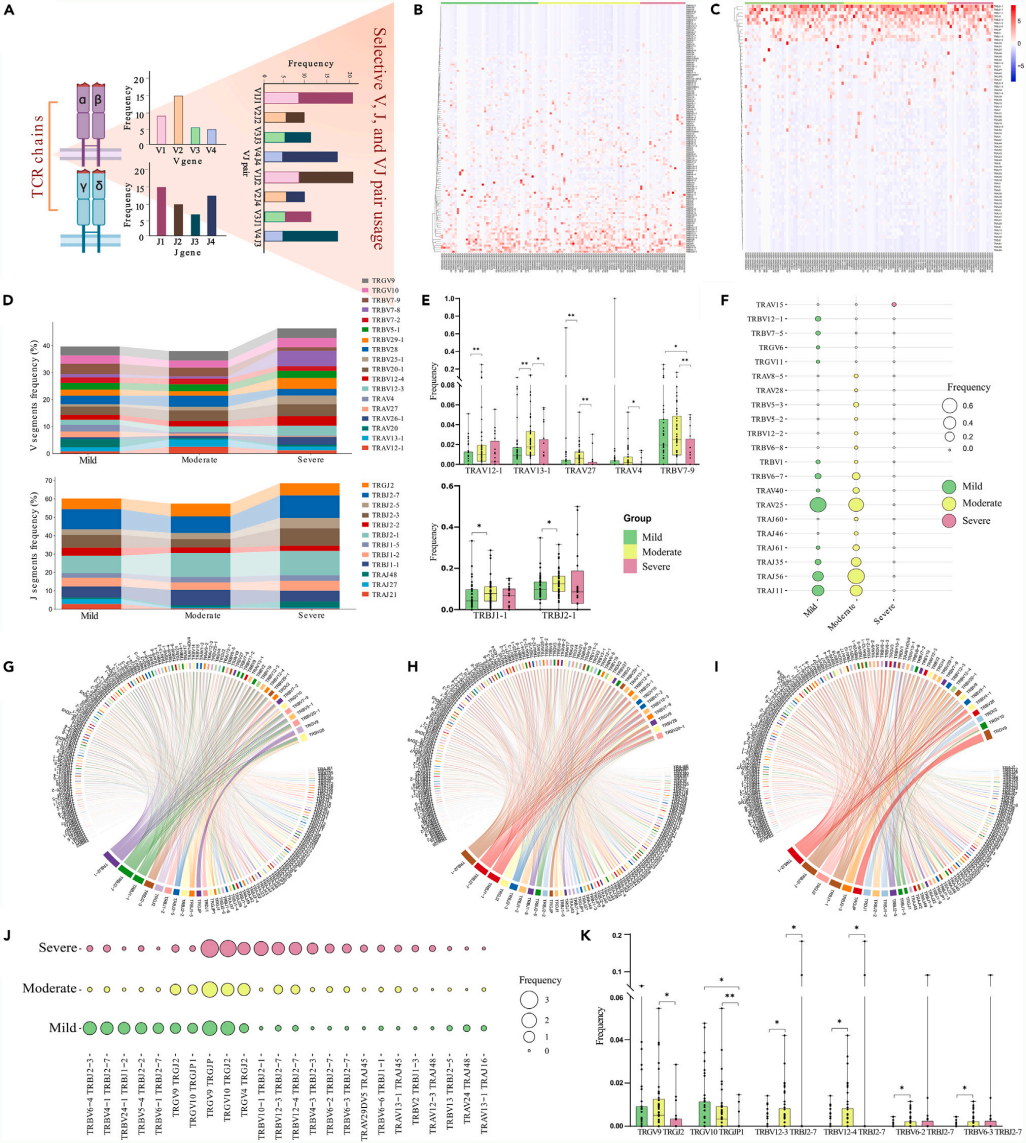
Gene usage analysis (V, J, and VJ pair segments) across dengue disease cohorts (A) Graphical representation showing the investigation of selective usage of V, J, and VJ pair segments. (B and C) Heatmaps showing frequency of (B) V segments, and (C) J segments across 108 patients segregated as mild, moderate, and severe. (D) Frequency distribution of the topmost V and J segments within all TCR chains across different dengue disease severity subgroups within the cohort. (E) Significant V and J segments among the topmost V and J segments identified in (D) using the Mann-Whitney U test (*p* < 0.05). (F) Frequency distribution of unique V and J segments either among one or two groups. (G–I) Circos plot summarizes the combination of all the VJ pairs across (G) Mild, (H) Moderate, and (I) Severe. (J) Frequency distribution of topmost VJ pairs within all TCR chains across different disease cohorts. (K) Significant VJ pairs among the topmost VJ pairs identified in (J). Data are represented as median +/− SEM. Mann-Whitney U test was utilized for the statistical significance (*p* < 0.05). Significance value is denoted as ∗, where ∗ indicates *p* ≤ 0.05, ∗∗ indicates *p* ≤ 0.01, ∗∗∗ indicates *p* ≤ 0.001 and ∗∗∗∗ indicates *p* ≤ 0.0001.

To determine if there is a preferential or biased utilization of V and/or J segments, we examined the involvement of V and J segments in clonotype patterns among the patients with dengue infection (Figure 3A). Our analysis revealed selective usage patterns of V and J segments across the patients with mild, moderate, and severe dengue (Figures 3B and 3C). We identified a total of 122 V and 74 J segments within the TCR receptor, with 107 V and 68 J segments being common across the severity groupings (Figure S3).

The heatmap visualization demonstrated distinct sets of V and J segments that were preferentially utilized during dengue infection. Figure 3D illustrates the frequency distribution of the topmost V and J segments within all the TCR chains across different disease severity within the cohort. Notably, TRAV and TRBV segments, followed by TRGV segments, exhibited increased read frequencies within the dengue severity groups. Specifically, *TRAV26-1*, *TRBV12-3*, *TRBV12-4*, *TRBV25-1*, *TRBV29-1*, and *TRBV7-8* were more frequently utilized in severe patients compared to the mild and moderate. Conversely, the frequencies of *TRAV20*, *TRAV27*, *TRAV4*, and *TRBV7-9* decreased with disease severity. Similarly, we observed a decrease in the frequencies of *TRAJ21*, *TRAJ27*, and *TRBJ2-2*, while *TRAJ48*, *TRBJ1-2*, *TRBJ2-1*, *TRBJ2-5*, and *TRBJ2-7* increased with worsening disease severity among the J segments. Additionally, consistent utilization of TRGV and TRGJ chains was noted across all disease severity groups, suggesting selective V and J segment usage during dengue infection. Figure 3E showcases the noteworthy V and J segments among the predominantly utilized segments across disease severity, identified through the Mann-Whitney test (*p* ≤ 0.05). All the significant V and J segments, including the topmost, are mentioned in Data S6.

We also found a few of the V and J segments that were uniquely present or shared among two of the groups (Figure 3F). Interestingly, *TRAV15* was uniquely present only in the severe patients, while it was absent in the mild and moderate. The usage of *TRAV25* and *TRAV40* was more pronounced in the mild and moderate patients, while it was absent in the severe. Also, a few of the TRBV segments (*12*-*1*, *7*-*5*) and TRGV segments (*6*, *11*) were present in the mild, while there was no observation of these in either moderate or severe. Some of the TRAJ segments (*11*, *35*, *56*, and *61*) were frequently used only in mild and moderate patients.

As both V and J segments represented selective usage patterns, we therefore looked for the frequencies of pairing segments. Figures 3G–3I depicts the frequency for each mild, moderate, and severe patient subgroup, respectively, in the form of circos plots. The topmost VJ pairing frequencies across all the severity groupings included *TRGV9/TRGJP*, where its frequency increased in the severe patients compared to mild and moderate. Many of the VJ pairs were observed to elevate with disease severity, including *TRGV10/TRGJ2*, *TRGV4/TRGJ2*, *TRBV10-1/TRBJ2-1*, *TRBV12-3/TRBJ2-7*, *TRBV12-4/TRBJ2-7*, *TRBV6-2/TRBJ2-7*, *TRBV6-3/TRBJ2-7*, *TRBV2/TRBJ1-3*, and *TRAV12-3/TRAJ48*. It was interesting to note that the *TRBJ2-7* segment, which showed increased frequency in the severe patients, was commonly paired with several TRBV segments, which resulted in an increase in the severity (Figure 3J). Also, a few of the VJ pairs exhibited reduced frequency with increased severity, including *TRBV6-4/TRBJ2-3*, *TRBV24-1/TRBJ1-2*, *TRBV5-4/TRBJ2-2*, *TRBV13/TRBJ2-5*, and *TRAV24/TRAJ48*. This suggests that the particular TRBV-TRBJ pair was selectively used and increased with dengue disease severity. The significant VJ pairs among the topmost utilized pairs across disease severity are highlighted in Figure 3K, as determined by the Mann-Whitney test (*p* ≤ 0.05) (Data S6).

### Differential length and amino acid composition of CDR3 regions in TCR chains correlate with dengue severity

**Figure 4:**
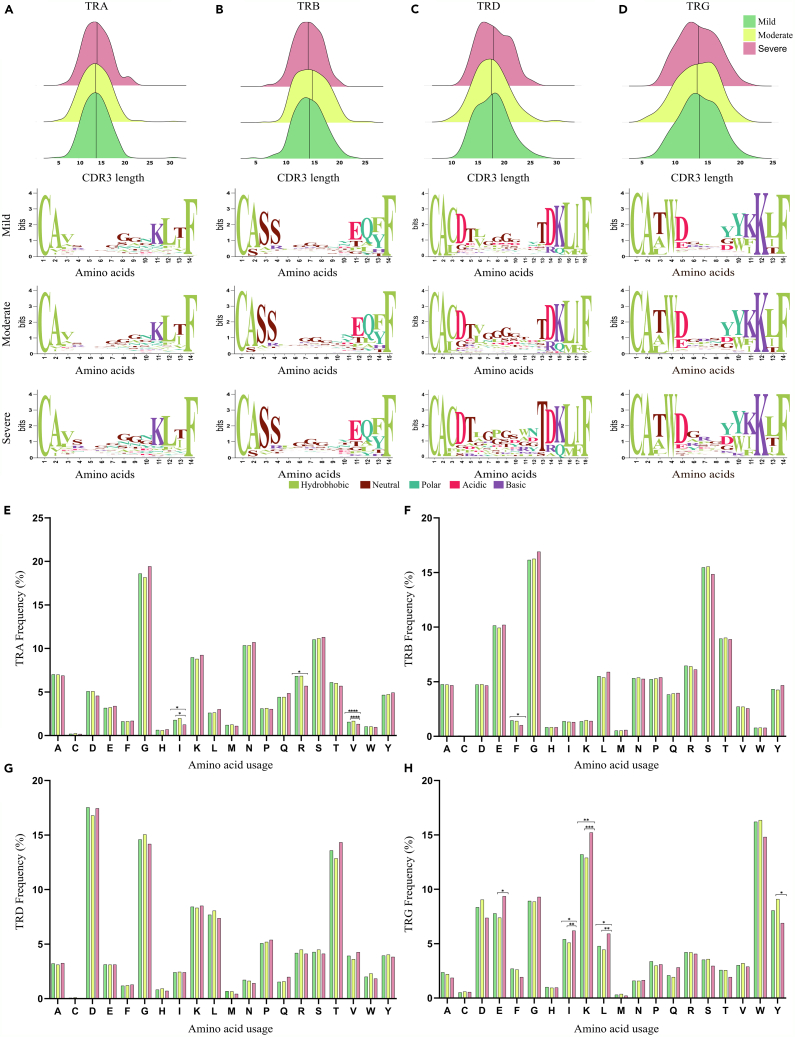
Differential CDR3 motifs and Changes of amino acid composition in dengue severity groupings (A–D) Distribution of CDR3 length and amino acid motifs within the (A) TRA, (B) TRB, (C) TRD, and (D) TRG, across the mild, moderate and severe subgroups. The Ridge plot (upper) shows the number of chains sequenced for a given length. We selected 14, 14, 18, and 14 amino acid lengths from each CDR3 of TRA, TRB, TRD, and TRG, respectively, and performed WebLogo analysis (lower). (E–H) CDR3 amino acid compositions. The bar charts depict the occurrence of individual amino acids in the CDR3 of TCR (E) TRA, (F) TRB, (G) TRD, and (H) TRG, across mild, moderate and severe. Significance of amino acids between the disease cohorts was performed using the Man-Whitney U test (*p* < 0.05). Significance value is denoted as ∗, where ∗ indicates *p* ≤ 0.05, ∗∗ indicates *p* ≤ 0.01, ∗∗∗ indicates *p* ≤ 0.001 and ∗∗∗∗ indicates *p* ≤ 0.0001.

The length and amino acid composition of the immunoglobulin CDR3 region affect antigen recognition, TCR repertoire diversity, clonal expansion, and immune escape mechanisms.^32^ Therefore, we focused on the length and amino acid distribution within all the TCR chains across mild, moderate, and severe patients to understand the dynamicity of the CDR3 region during dengue infection. α-CDR3 sequences had lengths ranging from 5 to 31 amino acids, with a median of 14 amino acids. β-CDR3 sequences had lengths ranging from 4 to 26 amino acids, with a median of 15 amino acids. γ-CDR3 sequences had lengths ranging from 6 to 22 amino acids, with a median of 14, while δ-CDR3 sequences had lengths ranging from 10 to 30 amino acids, although they had a longer median of 18 amino acids (Figures 4A–4D, upper panel). Interestingly, the δ-CDR3 length was longer than all the chains across all the comparison subgroups (Figure S4).

We also investigated whether the presence of any specific motif could provide prognostic information. The α-CDR3, β-CDR3, and δ-CDR3 sequences showed no strong over-representation of specific amino acids except for a slightly higher prevalence of glycine residues in the middle of the sequence logo. Interestingly, we observed an increased frequency of threonine (T) at positions 5 and 13 within the TRD of severe dengue patients. Also, at the 8 and 11 positions of δ-CDR3 in severe patients, the introduction of proline (P) and tryptophan (W) amino acids differentiates the CDR3 region among dengue severity (Figures 4A–4D, lower panel).

Previous studies have shown that the first and last three amino acid residues are buried and therefore are not directly involved in antigen binding. Therefore, we centered our focus on the surface exposed amino acids at positions 4–11 in α-CDR3, β-CDR3, and γ-CDR3, except δ-CDR3, where the loop region was considered between 4 and 15 positions. Based on this, we looked for the amino acid composition and plausible alterations in the CDR3 chains within the specified positions across dengue severity (Figures 4E–4H). Between the TCR chains, we observed that glycine (G) was mostly used in the TRA, TRB, and TRD. There was an increase in the usage of glycine in severe dengue patients across all the TCR chains, except for TRD. Serine (S), glutamic acid (E), and threonine (T) levels were elevated in the TRB chain; aspartic acid (D) and threonine (T) levels were increased in the TRD; while lysine (K), tryptophan (W), glutamic acid (E), and tyrosine (Y) levels were pronounced in the TRG chains. Interestingly, we observed a significant decrease in the hydrophobic amino acids, including isoleucine (I) and valine (V), within the TRA chain in the severe patients as compared to the mild and moderate groups. In contrast, the TRG chain showed an increase in the hydrophobic amino acids, including isoleucine (I) and leucine (L). Hydrophobic amino acids contribute to protein-protein interactions and membrane anchoring of TCRs.^33^ Increased hydrophobicity in TCRs can enhance epitope identification by improving the stability and specificity of the TCR-pMHC interaction.

### Strong positive correlation between dengue virus mutations and TCR chains highlights immune activation in severe patients

**Figure 5:**
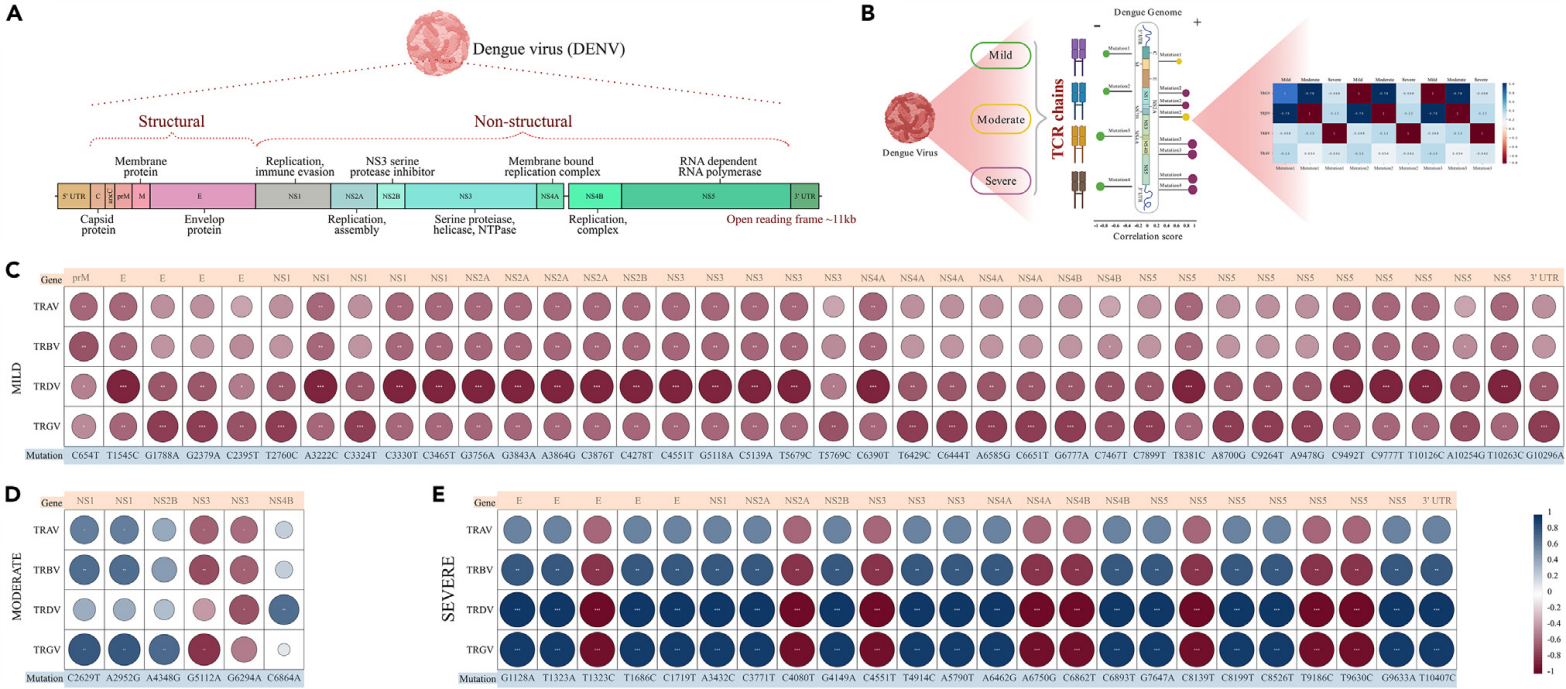
Significant correlation between dengue genome mutations and TCR chains across mild, moderate, and severe patients (A) Genome structure of DENV-2 (NC_001474). (B) Graphical representation of the TCR repertoire correlates with dengue genome mutation across the severity subgroups. (C–E) Correlation plots show the significant Pearson correlation analysis of different chains of TCR with mutation across the different genes of the dengue genome in mild (C), moderate (D) and (E) severe. Significance value is denoted as ∗, where ∗ indicates *p* ≤ 0.05, ∗∗ indicates *p* ≤ 0.01, ∗∗∗ indicates *p* ≤ 0.001 and ∗∗∗∗ indicates *p* ≤ 0.0001.

For a TCR to expand and proliferate, it must interact with a specific antigen. During dengue virus infection, the interaction of both structural and non-structural proteins with the host immune system is critical for inducing an active immune response, which in turn increases the proliferation of specific TCR clonotypes. However, this interaction can be significantly impacted by mutations within the viral genome, particularly those affecting key interacting proteins. Taking this into account, we conducted a detailed variant calling analysis across all the patients (112) in order to identify the presence of mutations in the dengue genome. Post holo-transcriptome analysis, 42 samples (mild = 19, moderate = 13, and severe = 10) revealed the presence of particular mutations across various positions within the DENV-2 genome. The DENV-2 genome coverage, serotype, along with sequencing read and depth information for these 42 dengue samples is listed in Data S7. The open reading frame (ORF) of the dengue genome is ∼11 kb, consisting of 3 structural (capsid (C), membrane (prM and M), and envelop (E), along with 7 non-structural (NS1, NS2A, NS2B, NS3, NS4A, NS4B, and NS5) proteins flanked by the untranslated regions at both the 5′ and the 3′ ends of the genome (Figure 5A). The mutation analysis identified 1504 mutation sites, with a gene-wise distribution highlighting the *NS5* gene as having the highest number of mutations and the 5′UTR the least.

We aimed to understand how these mutations correlate with the proliferation of TCR clonotypes, specifically focusing on different TCR chains (TRAV, TRBV, TRDV, and TRGV). For this, we employed Pearson correlation analysis between the mutation counts in each individual across the different severity subgroups and the count of TCR clonotypes within these groups (Figure 5B). Across mild, moderate, and severe patients, we identified 1407, 911, and 886 mutations, respectively. From these, 175, 9, and 40 mutations showed a significant correlation with TCR clonotype counts within different TCR chains (Data S8). Mutations that exhibited a strong correlation (r ≥ 0.7) were plotted to observe patterns across the mild, moderate, and severe patients, as shown in Figures 5C and 5D, and 5E, respectively. It was interesting to note that all the mutations in the mild patients negatively correlated with TCR chains, suggesting that mutations in this context might downregulate the immune response, possibly leading to less severe symptoms. The majority of mutations in the severe patients positively correlated with TCR chains, with a correlation coefficient (r) of up to 0.9. This indicates a potential upregulation or over-activation of TCR clonotypes, which could contribute to severe immune responses and cytokine storm characteristic of severe dengue.^15^ Another observation was significant and positive correlation of several mutations with the TRDV and TRGV chains within the severe patients. This might indicate that these mutations enhance the activation of γδ T cells, contributing to heightened immune responses. Although the maximum mutations were observed in the NS5 region that correlated with both TRDV and TRGV, one mutation, in particular *T10407C*, was positively and significantly correlated with both TRDV and TRGV in the severe patients only. The 3′UTR of the dengue virus plays a crucial role in the regulation of viral gene expression, RNA stability, and replication. It contains elements that interact with host cellular factors and machinery.^34^ The positive correlation suggests that certain elements or secondary structures within the DENV 3′UTR may be influencing host cellular processes that lead to an increased expression or activation of the TRD chain in T cells.

## Discussion

The composition and diversity of the TCR repertoire play crucial roles in shaping the immune response and influencing disease outcomes following viral infections.^35^ Previous studies have highlighted the dynamic alterations in the TCR repertoire upon several viral infections, such as influenza, Epstein-Barr virus (EBV), and SARS-CoV-2.^36^^,^^37^^,^^38^ Moreover, the expansion and persistence of specific TCRs during chronic cytomegalovirus (CMV) infection illustrate the dynamic and evolving nature of the TCR repertoire in response to viral infections.^39^ This suggests that upon viral infection, there is a dynamic alteration within the TCR repertoire, which underscores the importance of understanding TCR diversity and clonal dynamics vis-à-vis disease severity and clinical outcome. While dengue fever exhibits seasonal patterns, the challenge of multiple serotypes of dengue virus (DENV) impedes the development of a robust, lasting immune response capable of providing enduring protection against recurrent dengue infections.^40^

In this study, we have tried to shed light on the TCR repertoire dynamics during dengue infection. Using inferences from the 112 hospital admitted patients infected with DENV, we classified them as mild (45), moderate (46), and severe (21) on the basis of their total leukocyte and platelet counts. We observed that, along with leukopenia and thrombocytopenia, the severe dengue patients also showed a significant fall in neutrophils compared to the mild ones. This could be due to plasma leakage in severe dengue patients, which may result in the hemo-concentration and sequestration of the blood cells, including neutrophils,^10^ rather than circulating in the bloodstream, resulting in apparent neutropenia. Interestingly, while there was an overall decrease in total leukocyte counts with increasing dengue severity, we simultaneously noted a rise in lymphocyte counts, suggesting an increase in atypical lymphocytes. Previous research has linked higher counts of atypical lymphocytes with quicker recovery and reduced hospital stays.^28^ These atypical lymphocytes have been characterized as polyclonal CD19^+^ B cells, with their expansion particularly noted in severe dengue virus infections.^41^ The current theory suggests that these expanded lymphocytes initiate an antibody-mediated immune response against the dengue virus, explaining the significant increase in anti-dengue immunoglobulin G (IgG) antibodies, especially in cases of secondary dengue infections. Additionally, during DENV-2 infection, an expansion of CD8^+^ T cell subsets, notably the effector memory subset, has been observed.^42^ This uncontrolled and heightened immune response may also play a role in the pathogenesis of severe dengue.^43^

Through VDJ analysis of RNA-sequencing (RNA-seq) data using MiXCR and VDJtools of dengue-infected blood samples, we observed that across disease severity, moderate patients exhibited an increased number of TCR clonotypes compared to the mild and severe patients. In addition to this, the diversity indexes, indicating increased diversity of clonotypes, were also higher in moderate patients compared to the mild and severe. These findings suggest that the increased diversity and clonal expansion of TCR clonotypes in the moderate patients are possibly indicating increased heterogeneity among the dengue patients. Although both the number of clonotypes and diversity decreased in severe dengue patients, this might indicate a reduction in the TCR repertoire available for antigen recognition in severe dengue. It was interesting to note that the uniqueness within the TCR repertoire across all the subgroups, with the severe ones having no public clones, indicates dominant heterogeneity and diversity in the TCR repertoires within dengue patients. Decreased TCR clonotype and diversity indicate a limited repertoire of T cells available for antigen recognition, compromising the immune system’s ability to detect and respond effectively to a diverse range of pathogen-derived antigens. This reduction in TCR diversity limits immune flexibility, predisposing individuals to recurrent infections and disrupting immune regulation mechanisms, potentially exacerbating the severity of the infection.^20^^,^^44^^,^^45^

During T cell development, V(D)J recombination leads to the random combination of segments from gene families (V, D, J), generating a diverse array of potential TCR sequences.^46^ The resulting TCR sequences primarily form four types of TCR chains, with the α and β chains dominating while the γ and δ chains are less common.^47^ During infection, the T cells predominantly form α and β chain receptors, responding in an antigen-specific manner. However, a small fraction of γ and δ chain receptors also form, playing essential roles in initial immune and inflammatory responses.^48^^,^^49^ Nonetheless, their increase may indicate elevated inflammatory activity, potentially leading to a severe disease outcome.^50^ Our study observed an increased relative abundance of γ and δ chain receptors in severe dengue patients compared to the mild and moderate. This suggests that disease severity in dengue patients may be associated with an increased abundance of γ and δ TCR chain receptors.

The V and J gene segments play a crucial role in determining the antigen specificity and diversity of TCRs.^51^^,^^52^ Therefore, we investigated the distribution of V and J segments, along with VJ pairings, in dengue-infected individuals and their association with disease severity. We found several V and J segments exhibiting selective usage and differentiating severe dengue patients from mild and moderate patients. Specifically, the TRBV (*12*-*3*, *12*-*4*, *25*-*1*, *29*-*1*, and *7*-*8*) and TRBJ (*1*-*2*, *2*-*1*, *2*-*5*, and *2*-*7*) segments showed skewed usage in the severe patients compared to mild and moderate. Previous studies have also shown that individuals infected with DENV are relatively biased toward TRBV segment usage.^53^ This suggests that TRBV segments were selectively used in severe cases, indicating their association with increased disease severity. With maximum relative abundance across all the TCR chains, the TRB chain receptors, with their selective V and J segment usage, suggest their crucial involvement either in the progression or decline of dengue severity. V segments, which are variable regions involved in the V(D)J recombination process, are essential for generating TCR diversity and heterogeneity.^54^ This recombination event creates a vast array of TCRs, allowing the immune system to recognize a wide variety of pathogens.^55^ Therefore, the selective usage of V and J segments in response to specific pathogen challenges can provide critical insights into how TCR dynamics correlate with disease severity and immune response efficacy.

The CDR3 length can influence the affinity and avidity of TCR interactions with antigen-MHC complexes.^56^ T cells with TCRs possessing optimal CDR3 lengths for binding to specific antigenic peptides may undergo clonal expansion and selection during the course of an infectious disease.^57^ This expansion of T cell clones with appropriate CDR3 lengths is critical for mounting an effective immune response against the infecting pathogen.^58^ Our study reveals significant variations in the length of CDR3 regions among α, β, γ, and δ TCR chains in response to dengue infection severity, with δ-CDR3 sequences consistently longer than others across all severity groups. This finding suggests unique functional roles or structural adaptations of δ-chain TCRs in response to dengue virus antigens, potentially indicating enhanced antigen recognition capabilities or distinctive interactions with viral epitopes. These longer δ-CDR3 lengths likely play a crucial role in shaping immune surveillance and response efficacy during dengue infection.^59^

The amino acid composition within CDR3 regions showed significant differences linked to disease severity in dengue infection. Glycine (G) residues were notably prevalent across TRA, TRB, and TRD chains, with an increased presence observed in severe cases, except for TRD. This aligns with previous findings suggesting glycine’s role in enhancing TCR flexibility and antigen binding, crucial in the immune response against pathogens.^60^ Hydrophobic amino acids play a critical role in the structure and function of TCR proteins, influencing their interactions with peptide-major histocompatibility complex (pMHC) molecules and subsequent immune responses.^61^ In the context of dengue infection severity, the observed correlation between hydrophobic amino acid profiles in TCR CDR3 regions and disease severity highlights intriguing aspects of immune response modulation and potential clinical implications. The study’s findings indicate a decrease in the hydrophobic residues like isoleucine (I) and valine (V) in the TRA chain among severe dengue cases, contrasting with an increase in hydrophobic amino acids such as isoleucine (I) and leucine (L) in the TRG chain. This differential hydrophobicity suggests distinct functional roles for TCRs in responding to severe dengue infection. Hydrophobic amino acids are known to contribute to protein stability, membrane anchoring, and interactions with lipid bilayers or other proteins.^56^ In the context of severe dengue, where immune dysregulation and heightened inflammatory responses are characteristic,^62^ T cells with TCRs containing hydrophobic residues might be better equipped to recognize and respond to viral antigens efficiently. Therefore, the observed increase in hydrophobic residues in the TRG chain may reflect a need for enhanced stability or specific interactions crucial for TCR function during severe dengue pathology.^63^

During dengue virus infection, TCR expansion and proliferation are contingent upon interaction with specific antigens.^64^^,^^65^ The immune response is activated by both structural and non-structural proteins of the dengue virus, leading to an increase in the proliferation of specific TCR clonotypes.^66^ However, mutations within the viral genome, particularly those affecting key interacting proteins, can significantly influence this interaction. The study’s key finding of a strong positive correlation between DENV-2 mutations and TCR chains, particularly in severe patients, provides crucial insights into the potential interplay between viral evolution and immune response dynamics.^66^ By analyzing mutations across the dengue genome, notably within proteins like NS5, involved in viral replication and immune evasion, our study identifies specific mutations that significantly correlate with increased TCR clonotype counts, particularly in TRDV and TRGV chains associated with γδ T cells. This suggests that these mutations may enhance γδ T cell activation, potentially contributing to the intense immune responses characteristic of severe dengue, including cytokine storms.^67^ These findings align with previous studies indicating that viral mutations impacting antigen presentation can influence disease severity by modulating TCR recognition and subsequent immune activation pathways.^68^ The observed negative correlation between mutations and TCR clonotypes in mild patients suggests a potential mechanism where viral mutations dampen immune responses, potentially leading to less severe disease outcomes, as observed in other diseases, including COVID-19.^69^^,^^70^

### Limitations of the study

Despite the valuable insights provided by this study, several limitations must be acknowledged to contextualize the findings. First, the study’s sample size and geographical focus on a single outbreak in India may affect the generalizability of the results to other regions or outbreaks involving different dengue virus serotypes. Plasma leakage, recognized as an important determinant of dengue severity according to WHO (2009) guidelines, was not assessed in our study due to the lack of ultrasound data (not part of standard clinical management) or other clinical markers required for its detection. The inclusion of such data could have provided a more comprehensive evaluation of disease severity and potentially strengthened the correlation between clinical outcomes and the immune response dynamics we observed. Future studies in this direction would be taking this aspect into consideration. The presence of IgM and IgG levels would also have been crucial for distinguishing between primary and secondary infections. The lack of these data limits our ability to fully explore the impact of prior infections on disease severity and to draw more robust correlations between infection status and TCR repertoire dynamics. Future studies would be incorporating this information. Additionally, the temporal constraint of focusing solely on the 2022 outbreak limits the ability to observe long-term mutation trends and their effects on disease severity. The lack of a longitudinal study design to track TCR repertoire changes over time in individual patients also restricts our ability to identify dynamic shifts in TCR diversity and segment usage during the progression from mild to severe dengue. Moreover, while this study relied on bulk RNA-seq technology, incorporating functional assays such as cytokine profiling could provide deeper insights into how specific T cells and their respective TCRs contribute to protective or pathogenic immune responses. Integrating TCR-specific sequencing using cutting-edge single-cell high-throughput technologies and other omics approaches, including proteomics and metabolomics, could offer a more holistic view of the immune response to DENV infection. This multi-omics strategy can reveal interactions between TCR dynamics and other molecular pathways involved in disease progression. Addressing these limitations in future research would enhance the comprehensiveness of the findings and contribute to a robust understanding of the genetic factors influencing dengue virus pathogenicity, ultimately informing better public health strategies.

### Conclusion

This study delves into TCR dynamics in dengue infection, revealing a link between disease severity and immune response. We observed leukopenia, thrombopenia, and elevated lymphocyte levels as severity increased. A rich heterogeneity in TCR clonotypes suggests significant individual-specific TCR diversity among dengue patients. Notably, elevated γ and δ TCR chains in severe patients indicate their potential role in inflammation. Severe patients showed unique TRGV and TRBV segment usage and extended δ-CDR3 sequences, pointing to specialized functional roles of δ-chain TCRs. Additionally, mutations in the NS5 and 3′UTR regions of the dengue genome positively correlated with TRDV and TRGV chains, suggesting these mutations contribute to TCR chain abundance in severe infections. Overall, the findings underscore the importance of TCR diversity and specific segment usage in understanding dengue severity and immune response with potential of their usage as disease severity indicator.

## Resource availability

### Lead contact

Further information and requests for resources and reagents should be directed to and will be fulfilled by the lead contact, Rajesh Pandey (rajeshp@igib.in).

### Materials availability

This study did not generate new unique reagents and material.

### Data and code availability

• RNA-seq data have been deposited at NCBI SRA and are publicly available as of the date of publication. Accession numbers are listed in the key resources table. All the data reported in this paper will be shared by the lead contact upon request.
• This paper does not report the original code.
• The datasets presented in this study can be found online at the NCBI SRA under the BioProject accession number PRJNA1071729 and GISAID under the accession ID EPI_SET_240925yg https://doi.org/10.55876/gis8.240925yg.
• Any additional information required to reanalyze the data reported in this paper is available from the lead contact upon request.

## Acknowledgments

The authors gratefully acknowledge the participation of all dengue patients involved in this study. Special recognition is extended to Aanchal Yadav, Priti Devi, and Aparna Swaminathan for their contributions to sample isolation, library preparation, and sequencing. The authors also thank Dr. Bharti Kumari for her role as research manager and coordination with funders. Appreciation is extended to Anil Kumar and Nisha Rawat for their assistance in dengue sample transport and management. Special acknowledgment to Partha Chattopadhyay, whose invaluable assistance and support were instrumental for this study. K.K. acknowledges the 10.13039/501100001412CSIR for providing research fellowship support. This research was supported by the funding support from 10.13039/100000865Bill and Melinda Gates Foundation (grant no. INV-033578) and 10.13039/100000877Rockefeller Foundation (grant number—2021 HTH 018) to R.P.

## Author contributions

K.K.: conceptualization, methodology, formal analysis, investigation, data curation, visualization, and writing—original draft. S.Y.: formal analysis, visualization, writing—original draft. B.T.: resources. S.B.: resources. R.P.: conceptualization, methodology, supervision, writing—review and editing, funding acquisition. All authors contributed to the article and approved the submitted version.

## Declaration of interests

All the authors affirm that there is no conflict of interest while conducting the study. We also confirm that the funding body did not have any role in planning, execution and inferences drawn from the study.

## STAR★Methods

### Key resources table

**Table:** 

REAGENT or RESOURCE	SOURCE	IDENTIFIER
Blood RNA extraction	QIAmp RNA Blood mini kit, Qiagen	Cat. No. 52304
TruSeq® Stranded Total RNA Library Prep Globin	Illumina	Cat. No. 20020612
AMPure XP	Beckman Coulter	Cat. No. A63881
Agencourt RNAClean XP Kit	Beckman Coulter	Cat. No. A63987
Qubit dsDNA HS Assay kit	Symbio (Thermo Fisher Scientific)	Cat. No. Q32854
Agilent 2100 Bioanalyzer	Agilent	Cat. No. 5067-4626

**Deposited data**		

RNA-seq data	This study, NCBI Sequence Read Archive (SRA) database	SRA: PRJNA1071729; [GISAID]: [EPI_SET_240925yg] https://doi.org/10.55876/gis8.240925yg

**Software and algorithms**		

bcl2fastq	NA	GitHub - brwnj/bcl2fastq: NextSeq specific bcl2fastq2 wrapper.; RRID: SCR_015058
FastQC	Andrews S.^71^	Babraham Bioinformatics - FastQC A Quality Control tool for High Throughput Sequence Data; RRID: SCR_014583
Trimmomatic v0.39	Bolger et al.^72^	USADELLAB.org - Trimmomatic: A flexible read trimming tool for Illumina NGS data; RRID: SCR_011848
MiXCR	Bolotin et al.^73^	https://github.com/milaboratory/mixcr; RRID: SCR_018725
VDJtools	Shugay et al.^74^	https://github.com/mikessh/vdjtools
WebLogo	Crooks et al.^75^	https://weblogo.berkeley.edu/logo.cgi; RRID: SCR_010236
BWA-MEM	Vasimuddin et al.^76^	https://github.com/lh3/bwa; RRID: SCR_010910
SnpEff	Cingolani et al.^77^	https://pcingola.github.io/SnpEff/; RRID: SCR_005191
GraphPad Prism 9	NA	https://www.graphpad.com/; RRID: SCR_002798
Srplot	Tang et al.^78^	http://www.bioinformatics.com.cn/srplot
RAWGraphs	Mauri et al.^79^	https://app.rawgraphs.io/
R 4.3.2	NA	https://www.r-project.org/; RRID: SCR_001905

### Experimental model and study participant details

#### Participant demographics and sample collection

The study aimed to investigate the composition, diversity, clonal expansion, and dynamic changes within the TCR repertoire during the differential disease severity of dengue. Blood samples were drawn from 112 hospital admitted patients who tested positive for dengue using NS1 antigen testing, collected during the peak dengue season from August to November 2022. After obtaining written consent, 2–3 mL blood samples were taken in EDTA vials by the paramedical team at MAX Super Speciality Healthcare Hospital in New Delhi, India. All necessary clinical information was retrospectively collected by meticulously reviewing the electronic medical records of each participant. The institutional ethical clearance was duly obtained for this study from both CSIR-IGIB and Max Super Speciality Hospital. The research was thoroughly reviewed and approved by the CSIR-IGIB Human Ethics Committee (Ref No: CSIR-IGIB/IHEC/2020-21/01). The study was conducted in strict adherence to the ethical principles outlined in the Declaration of Helsinki.

### Method details

#### Nucleic acid extraction

For the extraction of RNA from blood samples, the Qiagen QIAmp RNA Blood Mini Kit was used with modified protocol. This involved a 5-min lysis incubation period followed by centrifugation, as well as 2–3 min wash incubations to ensure efficient extraction of total RNA. Subsequently, the purity of the isolated RNA was assessed using a NanoDrop spectrophotometer and gel electrophoresis, ensuring high-quality RNA suitable for downstream next generation sequencing (NGS) application. The extracted RNA was then stored at −80°C to maintain its integrity until further processing for RNA-seq library preparation.

#### Library preparation and sequencing

Libraries were prepared using 250 ng of total RNA with the Illumina TruSeq Stranded Total RNA Library Prep Globin kit (Illumina, Cat. No. 20020612). Initially, globin mRNA and both cytoplasmic and mitochondrial ribosomal RNA, which are abundant in whole blood, were selectively removed. Post fragmentation, the RNA fragments were subjected to first-strand cDNA synthesis using reverse transcriptase and random primers. Subsequently, double-stranded cDNA was generated with DNA polymerase I and RNase H, followed by purification using AMPure XP beads (Beckman Coulter, A63881). Adenylation of the 3ʹ blunt ends of the double-stranded cDNA was performed, and sequencing libraries were uniquely indexed and enriched through PCR amplification.

Library quality was assessed using Agilent 2100 Bioanalyzer for fragment size analysis and the Qubit dsDNA HS assay kit (Thermo Fisher Scientific; catalog no. Q32854) for concentration determination. Libraries were diluted to 4 nM and pooled in equimolar amounts, with each pool containing 24 samples. Paired-end sequencing with 2 × 151 read length was conducted on Illumina NextSeq 2000 platform at a final loading concentration of 650 p.m.

#### Pre-processing of raw sequencing data

We utilized transcriptome sequencing data in FASTQ format of DENV-2 for the bulk TCR repertoire analysis. Initial quality assessment was conducted using FastQC^71^ to identify reads with low quality (phred score <20) and adapter contamination, which were subsequently trimmed using Trimmomatic v.0.39.^72^ Following these preprocessing steps to ensure high-quality sequencing data, we employed MiXCR software^73^ for TCR clonotype identification, facilitating the conversion from raw sequences to quantitative clonotype data. The MiXCR analysis pipeline involved aligning sequencing reads to the V, D, J, and C genes of T cell receptors from the IMGT (international ImMunoGeneTics) database. Subsequently, the aligned reads were assembled to extract CDR3 gene regions, crucial for TCR diversity analysis. Finally, identified clonotypes were exported as “exportClones” for each sample. To facilitate further analysis, we utilized VDJtools^74^ to convert MiXCR’s output into a text file formatted for VDJtools, enabling comprehensive downstream analysis of TCR repertoire dynamics and diversity.

#### Analysis of CDR3 sequences and diversity estimation

Different analysis, such as basic stats, CDR3 length profiles and V(D)J segment usages, Pool and Join Amino acids, richness and evenness, and identification of specific motifs based on CDR3 sequences, were done by using VDJtools with default parameters. We also estimated TCR clonal diversity using several parameters, including chao1, d50, Shannon-Wiener, and inverse Simpson diversity, that provide insights into clonotype abundance, richness (number of unique clones), and evenness (distribution of clonotype frequencies within the sample). For the TCR chain distribution, the relative abundance of each TCR chain (TRAV, TRBV, TRDV, and TRGV) was calculated across the severity groupings and statistically tested using the Fisher exact test.

#### Gene usage analysis

Comparative analysis of V, J, and VJ segment usage was performed based on their read frequencies in each segment/pair within each sample and compared across the dengue severity subgroups. For identifying the statistically significant segments, a Mann-Whitney U test was performed on the frequencies of the V and J genes, and the VJ pairing. A sequence logo is a graphical representation of amino acids or nucleotides at each position within a sequence alignment, which is generated using the WebLogo tool.^75^ It illustrates the frequency and significance of each character at every position, aiding in the identification of conserved motifs and functional domains in proteins or regulatory elements in nucleic acids.

#### Mutation analysis

Raw reads unmapped to human genomes were aligned against the DENV-2 genome (NC_001474) using BWA-MEM,^76^ resulting in BAM files. Variant calling was then conducted using bcftools to identify differences between the sequenced dengue virus genomes and the reference genomes. The raw variant calls were filtered to remove low-confidence calls, ensuring only high-quality variants were retained. The filtered variants were then annotated using SnpEff,^77^ a tool that adds biological context to the identified variants by predicting whether the variant is synonymous or non-synonymous or affects a splice site.^80^ Pearson correlation analysis was performed between all the mutation and TCR chain counts across the dengue severity subgroups to observe the strong and significant correlations.

### Quantification and statistical analysis

Statistical significance was calculated using the two-tailed Mann-Whitney U test, Fisher’s exact, and Chi-square tests. The statistical analysis was performed in the licensed version of GraphPad Prism 9. For data visualization, a combination of several tools and packages, including VDJtools, RAWGraphs, SRplots, WebLogo, GraphPad Prism 9, and R version 4.3.2, was utilized. All the statistical tests with *p* value < 0.05 were considered significant.

## Supplemental information

Document S1. Figures S1–S4

Data S1. Distribution of clinical symptoms in mild, moderate, and severe dengue positive patients

Data S2. Total sequencing reads with TCR aligned reads for 112 dengue samples segregated based on severity

Data S3. TCR clonotype distribution across mild, moderate, and severe groupings of dengue positive (108) patients with statistical analysis

Data S4. TCR clonal diversity indices across mild, moderate, and severe severity groupings in dengue positive patients with statistical significance

Data S5. Relative abundance & Fisher’s exact test of TCR repertoire chains across mild, moderate, and severe groupings of dengue positive patients

Data S6. Mann Whitney test for 122 V, 74 J segments and topmost VJ pair usage between mild, moderate, and severe groupings in dengue positive patients

Data S7. Dengue sample-specific serotype and genome coverage

Data S8. Pearson correlation between gene wise mutation in dengue genome and TCR chains across mild, moderate, and severe groupings
