# Supplementary material for: TCR repertoire dynamics and their responses underscores dengue severity
Source: iScience. 2024 Sep 16;27(10):110983. doi: 10.1016/j.isci.2024.110983 (PMC11472631; doi:10.1016/j.isci.2024.110983)
Supplement: Document S1. Figures S1–S4 [file mmc1.pdf]

iScience, Volume *27*

Supplemental information

TCR repertoire dynamics and their responses underscores dengue severity

Kriti Khare, Sunita Yadav, Bansidhar Tarai, Sandeep Budhiraja, and Rajesh Pandey

Supplemental information

Supplementary Figures:

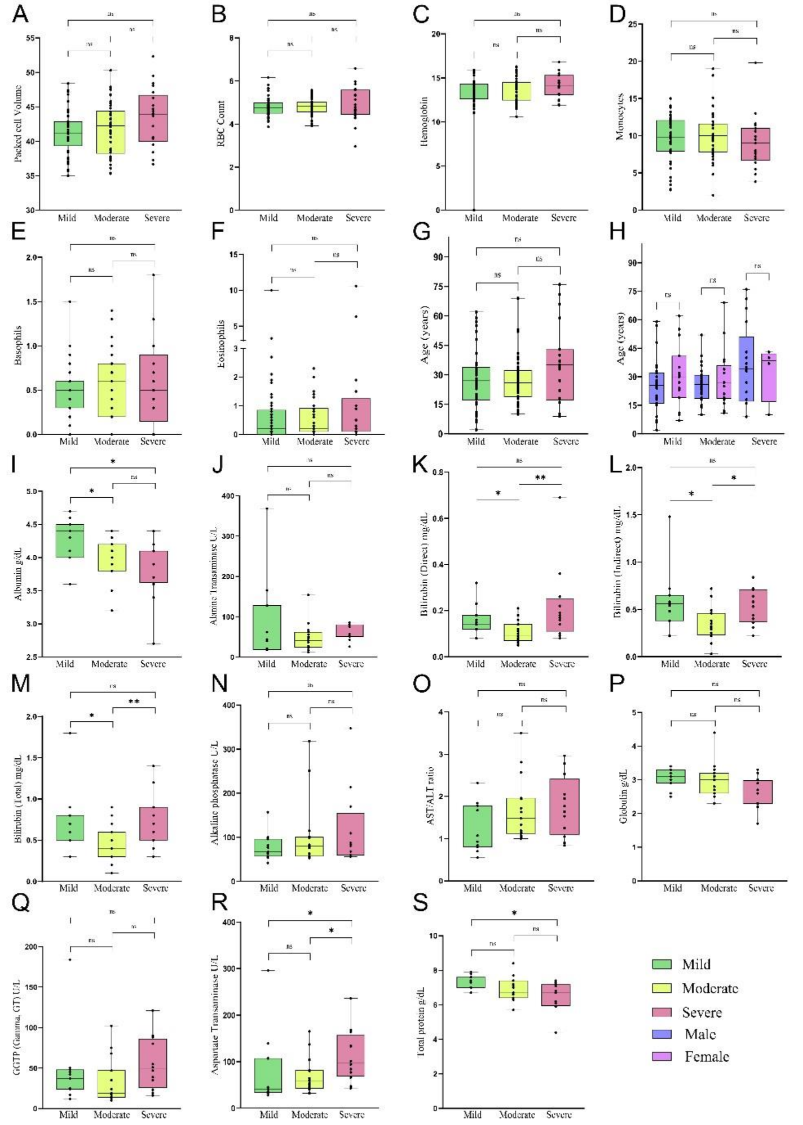

Supplementary Figure S1. Clinical and demographic parameters of the dengue severity groupings including, (A) Packed Cell Volume (%), (B) RBC Count (10^{12}/L), (C) Hemoglobin (g/dl), (D) Monocytes (%), (E) Basophils (%), (F) Eosinophils (%), (G) Age (years), (H) Gender, (I) Albumin (g/dL), (J) Alanine Transaminase (ALT) (U/L), (K) Bilirubin (Direct) mg/dL, (L) Bilirubin (Indirect) mg/dL, (M) Bilirubin (Total) mg/dL, (N) Alanine phosphatase (U/L), (O) AST/ALT ratio, (P) Globulin (g/dL), (Q) GGTP (Gamma, GT) (U/L), (R) Aspartate Transaminase (AST) (U/L), and (S) Total Protein (g/dL). Significance value is calculated by Mann-Whitney Utest and is denoted as $*$, where $*$ indicates $p \leq 0.05$, $**$ indicates $p \leq 0.01$, $***$ indicates $p \leq 0.001$ and $****$ indicates $p \leq 0.0001$. The non-significant values are denoted as “ns”.

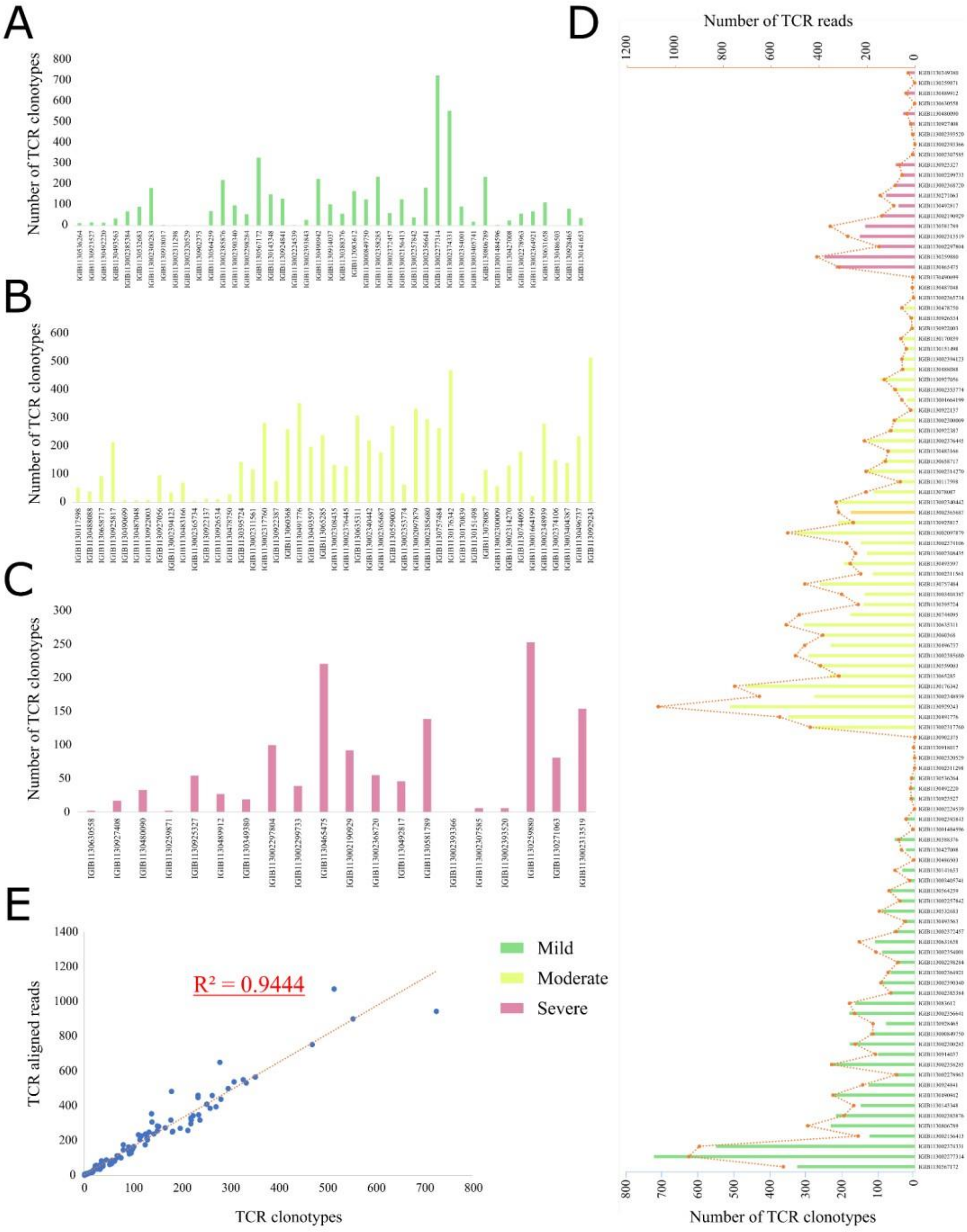

Supplementary Figure S2. Distribution of number of TCR clonotypes and reads. (A-C) Bar chart depicting the distribution of total number of TCR clonotypes in (A) mild, (B) moderate, and (C) severe dengue patients. (D) Correlation between total number of TCR clonotypes and number of aligned sequencing reads. (E) Linear and strong correlation ($R^2 = 0.9$) between the number of TCR clonotypes and aligned reads.

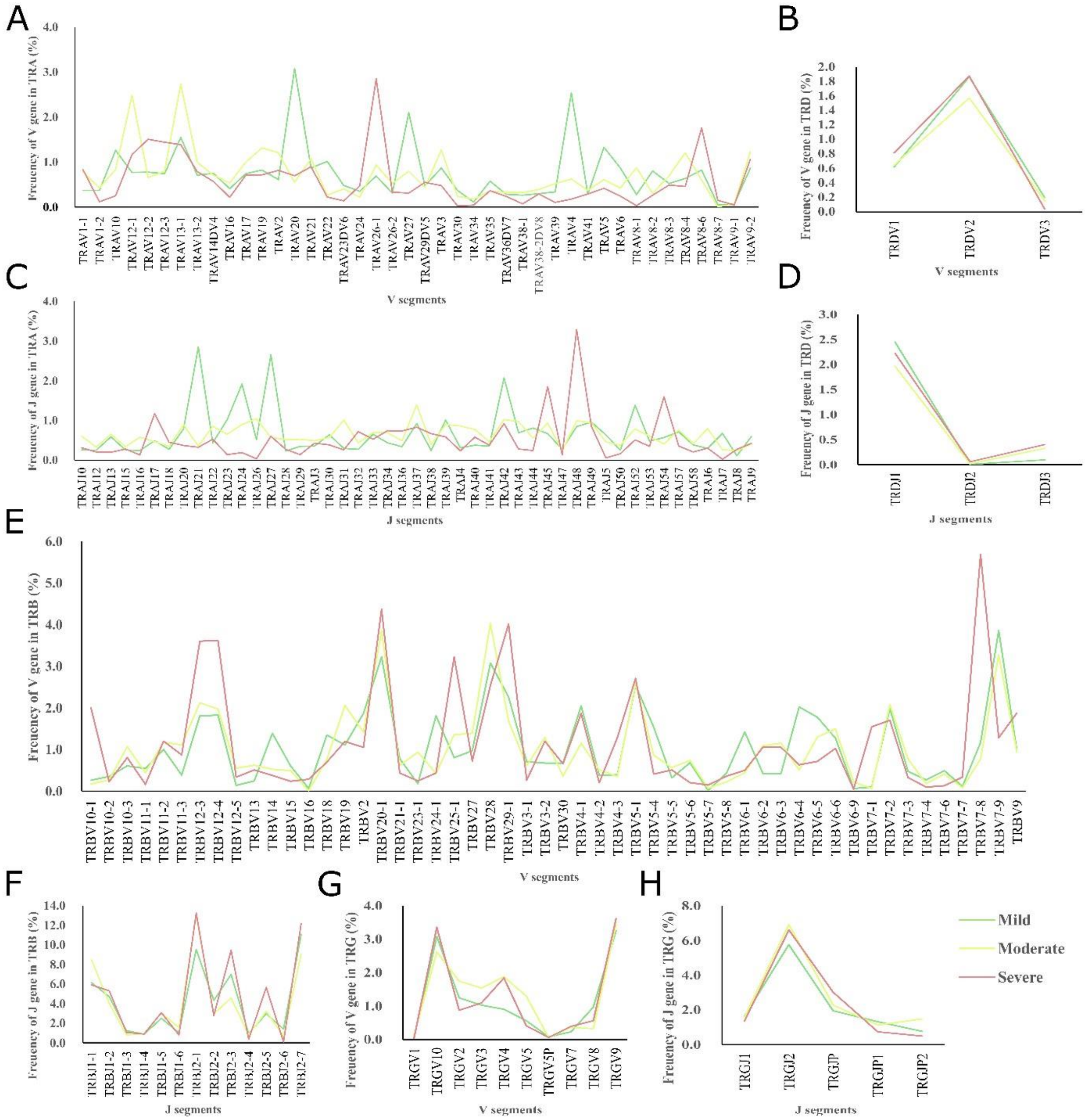

Supplementary Figure S3. Frequency of V and J gene usage across TCR chains. Frequency of (A) V gene in TRA, (B) V gene in TRD, (C) J gene in TRA, (D) J gene in TRD, (E) V gene in TRB, (F) J gene in TRB, (G) V gene in TRG, and (H) J gene in TRG. Mild, moderate, and severe are colored as green, yellow, and pink, respectively.

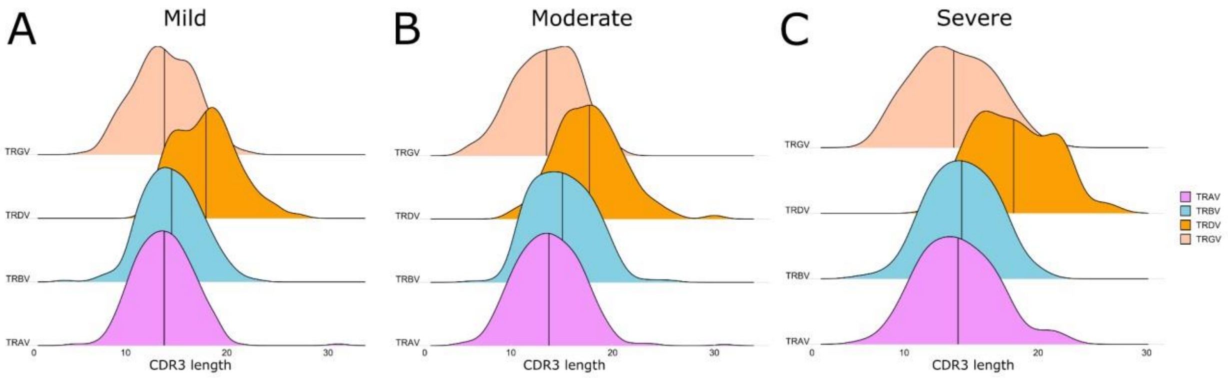

Supplementary Figure S4. CDR3 length of TCR chains in dengue disease severity. Length of CDR3 chain for TRAV, TRBV, TRDV, and TRGV across (A) mild, (B) moderate, and (C) severe dengue patients.
